# Automatic Super-Surface Removal in Complex 3D Indoor Environments Using Iterative Region-Based RANSAC

**DOI:** 10.3390/s21113724

**Published:** 2021-05-27

**Authors:** Ali Ebrahimi, Stephen Czarnuch

**Affiliations:** 1 Department of Electrical and Computer Engineering, Faculty of Engineering and Applied Science, Memorial University of Newfoundland, St. John’s, NL A1C 5S7, Canada; sczarnuch@mun.ca; 2 Discipline of Emergency Medicine, Faculty of Medicine, Memorial University of Newfoundland, St. John’s, NL A1C 5S7, Canada

**Keywords:** RANSAC, point cloud, bounding surface removal, wall removal, 3D background subtraction, 3D plane segmentation, 3D preprocessing technique, 3D size reduction

## Abstract

Removing bounding surfaces such as walls, windows, curtains, and floor (i.e., super-surfaces) from a point cloud is a common task in a wide variety of computer vision applications (e.g., object recognition and human tracking). Popular plane segmentation methods such as Random Sample Consensus (RANSAC), are widely used to segment and remove surfaces from a point cloud. However, these estimators easily result in the incorrect association of foreground points to background bounding surfaces because of the stochasticity of randomly sampling, and the limited scene-specific knowledge used by these approaches. Additionally, identical approaches are generally used to detect bounding surfaces and surfaces that belong to foreground objects. Detecting and removing bounding surfaces in challenging (i.e., cluttered and dynamic) real-world scene can easily result in the erroneous removal of points belonging to desired foreground objects such as human bodies. To address these challenges, we introduce a novel super-surface removal technique for 3D complex indoor environments. Our method was developed to work with unorganized data captured from commercial depth sensors and supports varied sensor perspectives. We begin with preprocessing steps and dividing the input point cloud into four overlapped local regions. Then, we apply an iterative surface removal approach to all four regions to segment and remove the bounding surfaces. We evaluate the performance of our proposed method in terms of four conventional metrics: specificity, precision, recall, and F_1_ score, on three generated datasets representing different indoor environments. Our experimental results demonstrate that our proposed method is a robust super-surface removal and size reduction approach for complex 3D indoor environments while scoring the four evaluation metrics between 90% and 99%.

## 1. Introduction

In image processing, background subtraction is widely used in object detection and tracking approaches [1,2,3] with broad applications such as human tracking [4,5], face recognition [6], traffic management [7], and surveillance systems [8,9]. Background subtraction is typically a preprocessing phase used to identify and differentiate the foreground pixels (representing objects of interest) from the background pixels (representing uninteresting information). The background pixels can then be subtracted or removed from the original image, leaving only the foreground pixels, which reduces the storage space requirements, reduces the computational complexity, and improves the overall algorithm performance of downstream image processing techniques. Established 2D background subtraction approaches are based on static background segmentation methods [10], adaptive Gaussian mixture models [11,12], real-time codebook models [13,14], and independent component analysis-based techniques [15,16]. Although advanced 2D background subtraction techniques can handle gradual illumination changes and repetitive movements in the background, they perform poorly in the presence of shadows or foreground regions with colors similar to the background [17]. The release of commercially available and inexpensive depth sensors such as the Microsoft Kinect opened new doors for improved background subtraction techniques because of the availability of additional depth data associated with each pixel of color data. Depth sensor (RGB-D) systems are more robust for background detection problems compared to classic color-based systems because depth data are largely invariant to color, texture, shape, and lighting [18,19]. As /a result of the advantages of combined depth and color information, data from RGB-D sensors have also been widely used in background segmentation methods (e.g., [17,20,21]) which have been thoroughly reviewed (see [22] for a comprehensive review of different 3D background subtraction methods and their capabilities in solving common background segmentation challenges).

Background subtraction methods are generally used to analyze individual images and identify the foreground by first estimating a reference background that is developed from historical information obtained from videos or sequences of images. Therefore, the classic application of 2D background subtraction is separating dynamic or moving objects from a relatively static or slow-changing background scene. However, RGB images only contain intensity information and spatial information that is largely restricted to the two dimensions of the image that are perpendicular to the camera’s perspective. Accordingly, identifying the boundaries between objects, or conversely identifying object interactions or contact, is limited mainly to detectable intensity differences. In applications that utilize RGB-D data, interactions or contact between objects and object spatial relationships can be more directly measured.

Furthermore, registration between depth and RGB data allows traditional 2D background subtraction approaches to be supplemented with additional depth-based approaches (e.g., [23,24]), and further allows RGB-D data to be represented as 3D point clouds [25]. For example, reliably identifying and removing static background components such as roads and walls before modeling the background can result in both improved background subtraction and improved foreground segmentation using both 2D and RGB-D data; improvements that to our knowledge have only been realized through approaches that require additional data and computation, such as motion estimation between consecutive frames (e.g., [26]). Identifying static background components suffers from the same limitations as modeling the entire background using 2D data, suggesting that little benefit is afforded by first removing these background objects, then modeling the background. However, with RGB-D data, parametrically modeled objects (e.g., planes, spheres, cones, cylinders, and cubes) are far more reliably detectable. As a result, researchers have attempted to segment or remove large planar surfaces (e.g., walls, ceiling, and floor surfaces) as a preprocessing or fundamental step before all other algorithms (e.g., [27,28,29]).

In general, large planar surfaces comprise a large percentage of points within each frame of RGB-D data captured in indoor environments. However, outside specific applications that seek to identify significant surfaces (e.g., ground plane detection [30]), large planar surfaces are not often the objects of interest in 3D computer vision applications. Notably, smaller planar surfaces (e.g., tabletops, chair seats and backs, desks) are more likely to be of interest than larger surfaces at the boundaries of the scene. Furthermore, the large bounding surfaces can decrease the performance of 3D computer vision algorithms (e.g., object segmentation and tracking) by cluttering their search space. Therefore, a robust removal technique for points that belong to surfaces at the outer boundaries of the RGB-D data can significantly reduce the search space and bring three main benefits to the computer vision systems: improving downstream results, speeding up downstream processes, and reducing the overall size of the point clouds. We refer to these large surfaces, which may include points from multiple planes or objects (e.g., points that represent a wall, window, and curtains) at the extremes of the point clouds as super-surfaces in the context of background subtraction applications. Our objective is to develop a robust technique of removing super-surfaces from RGB-D data captured from indoor environments and represented as point clouds. Our intention is that our super-surface removal technique will function as a pre-processing step, improving existing computer vision techniques, and reducing storage requirements, without removing any foreground data.

### 1.1. Related Work

Point cloud data are classified as either organized or unorganized datasets. Organized datasets are represented by a matrix-like structure, where the data (i.e., voxels) are accessible by index, usually according to their spatial or geometric relationship. Unlike organized point clouds, the relationships between adjacent voxels of unorganized datasets are unknown, and the data are simply stored as a one-dimensional, unsorted array. Data from RGB-D sensors are typically stored as organized point clouds, where indexes are referenced according to the spatial resolution of the sensor. However, point cloud pre-processing steps such as down-sampling often produce unorganized point clouds. While it is trivial to convert an organized to an unorganized point cloud, the converse is much more complicated and costly. Since spatial relationships between voxels are preserved, plane detection is less challenging for organized point clouds. However, computer vision approaches designed for unorganized datasets are universal [31] (i.e., they can also be used for organized datasets), so a robust plane detection approach must work with unorganized datasets and not rely on the spatial relationship of voxels as derived from their storage indices. In general, plane segmentation methods can be categorized into three categories: model fitting-based methods, region growing-based methods, and clustering feature-based methods.

#### 1.1.1. Model Fitting-Based Methods

Random Sample Consensus (RANSAC) [32] and Hough transform [33] are the most commonly used model fitting-based methods for plane segmentation. The Hough transform is a voting technique for identifying objects that can be modeled parametrically, such as lines, planes, and spheres. Every point is transformed into a unique function (e.g., a sinusoid when modeling lines) in a discretized parameter space. Objects of interest can then be extracted by selecting the maximal intersections between the functions in the discretized parameter space, where the spatial tolerance for model fitting (e.g., to compensate for sensor resolution, noise, and object surface variations) can be accommodated by changing the resolution of the parameter space. The Hough transform has been successfully used for 3D plane segmentation in several publications (e.g., [34,35]). Unfortunately, although Hough transform-based methods can robustly segment 3D objects, they necessitate large amounts of memory and significant computational time [36], and their results depend significantly on the proper selection of segmentation parameters [37]. More importantly, Hough transform is unable to discriminate between voxels that lie within a parameterized model (i.e., inliers) and outside the model (i.e., outliers) since spatial relationships are not preserved in the Hough parameter space. The result is that foreground points that belong to objects that are spatially close to the parameterized background model will often be associated with the model, and ultimately the background scene.

The RANSAC algorithm begins with a random selection of data points that estimate the corresponding model parameters (e.g., three points for a plane). Then, the remaining points are examined to determine how many of them are well-approximated by the model. Terminally, the RANSAC algorithm returns the model with the highest percentage of inliers that are within a fixed threshold (e.g., the orthogonal distance from the planar model). Many researchers have proposed RANSAC-based algorithms for 3D plane segmentation, such as [37,38,39].

Awwad et al. [37] proposed a RANSAC-based segmentation algorithm that first clusters the data points into small sections based on their normal vectors and then segments the planer surfaces. This implementation of RANSAC prevents the segmentation of spurious surfaces in the presence of parallel-gradual planes such as stairs. Chen et al. [38] developed an improved RANSAC algorithm through a novel localized sampling technique and a region growing based approach. Their proposed method, intended to segment polyhedral rooftops from noisy Airborne Laser Scanning (ALS) point clouds, is based on the assumption that rooftops comprise only planar primitives. Li et al. [39] proposed an enhanced RANSAC algorithm based on Normal Distribution Transformation (NDT) cells to prevent segmenting spurious planes. The algorithm considers each NDT cell rather than each point. After dividing the data points into a grid of NDT cells, a combination of the RANSAC algorithm and an iterative reweighted least-square approach fit a plane in each cell. Finally, a connected-component approach extracts large planes and eliminates points that do not belong to planes. Although the proposed method can detect 3D planes more reliable and faster than the standard RANSAC, it requires cell size tuning for different datasets.

According to a performance comparison by Tarsha-Kurdi et al. [40], the RANSAC algorithm outperforms the Hough transform approach for 3D roof plane segmentation in terms of both speed and accuracy. However, RANSAC suffers from spurious plane detection in complex 3D indoor environments [39].

#### 1.1.2. Region Growing-Based Methods

In general, region growing-based methods have two main stages. First, they pick a seed point and then merge the neighboring points or voxels that comply with the predefined criteria (e.g., similar normal vector). Several researchers proposed point-based, voxel-based, and hybrid region growing techniques for 3D point cloud segmentation. Tóvári and Pfeifer [41] proposed a point-based region growing algorithm that merges adjacent points to a seed region based on their normal vectors and distance to the adjusting plane. Nurunnabi et al. [42] utilized the same criteria but with a different seed point selection approach and a better normal vectors estimation.

Voxel-based region growing algorithms (e.g., [43,44]) improve the speed and robustness of point-based methods by voxel-wise processing of the 3D point clouds. Xiao et al. [45] proposed a 3D plane segmentation method based on a hybrid region growing approach utilizing a subwindow and a single point as growth units. Although their technique is significantly faster than the point-based region growing approach, it was intended for only organized point clouds. Vo et al. [36] proposed a fast plane segmentation technique for urban environments. The method consists of two main stages: first, a coarse segmentation was achieved using an octree-based region growing approach, then a point-based process refined the results by adding unassigned points into incomplete segments.

Region growing-based methods are easy to employ for 3D plane segmentation, primarily for organized point clouds. However, their output results depend on the growing criteria, the seed point selection, and the textures or roughness of planes [46]. Furthermore, they are not robust to occlusion, point density variation, and noise [47].

#### 1.1.3. Clustering Feature-Based Methods

Clustering feature-based methods adopt a data clustering approach based on characteristics of planar surfaces, such as normal vector attributes. Filin [40] proposed a clustering method based on an attribute vector comprising a point location, its tangent plane’s parameters, and the height difference between the point and its adjacent points. In another work, Filin and Pfeifer [41] computed the point features using a slope adaptive neighborhood system and employed a mode-seeking algorithm to extract clusters. Then, they extended or merged those clusters with their adjacent points or clusters if they share analogous standard deviations and surface parameters. Czerniawski et al. [21] applied a simple density-based clustering algorithm to a normal vector space (i.e., a Gaussian sphere). The dense clusters on the Gaussian sphere represent the directions perpendicular to large planes. Zhou et al. [42] proposed a clustering feature-based method for segmenting planes in terrestrial point clouds. First, they created a 4D parameter space using planes’ normal vectors and their distance to the origin. Then, they segmented the planer surfaces by applying the Iso cluster unsupervised classification method.

Despite the efficiency of clustering feature-based methods, employing multi-dimensional features in large point clouds is computationally intensive [28]. Furthermore, they are sensitive to noise and outliers [43]. Moreover, the clustering segmentation approaches cannot reliably segment the edge points as these points may have different feature vectors compare to the surface points.

### 1.2. Contributions

Several 3D plane segmentation methods can satisfactorily detect different planar surfaces for various computer vision applications. However, to our knowledge, no approaches have been developed specifically for bounding surface removal, particularly in complex environments: environments that are cluttered, and where the placement of a depth sensor is not ideal. Additionally, existing segmentation approaches generally segment foreground points that belong to parametrically modeled objects of interest (e.g., planes, spheres, cones, cylinders, and cubes), rather than with the intention of removing background points belonging to the bounding surfaces. Therefore, existing approaches can easily remove critical foreground objects (or portions of foreground objects), significantly impacting the segmentation accuracy of semantic information. To overcome these limitations, we propose a method of removing background bounding surfaces (i.e., super-surfaces, such as walls, windows, curtains, and floor). Our novel method is particularly suited to more challenging and cluttered indoor environments, where differentiating between foreground and background points is complicated. Accordingly, our objective is to develop a robust background super-surface removal method that can support a wide range of sensor heights relative to the ground (i.e., support varied sensor perspectives) for organized and unorganized point clouds. Additionally, our approach must ensure that foreground objects, and points belonging to those objects, are preserved during super-surface removal.

Our method significantly reduces the search space, and it can considerably reduce the size of 3D datasets, depending on the number and size of the super-surfaces in each point cloud. Furthermore, when used as a preprocessing step, our approach can improve the results and the running time of different 3D computer vision methods such as object recognition and tracking algorithms. The remainder of this paper is organized as follows. In the next section, we describe our proposed 3D super-surface removal method. In Section 3, we provide our experimental results and the evaluation of our proposed method. In Section 4, we present our discussion and future work, followed by conclusions in Section 5.

## 2. The Iterative Region-Based RANSAC

**Figure 1:**
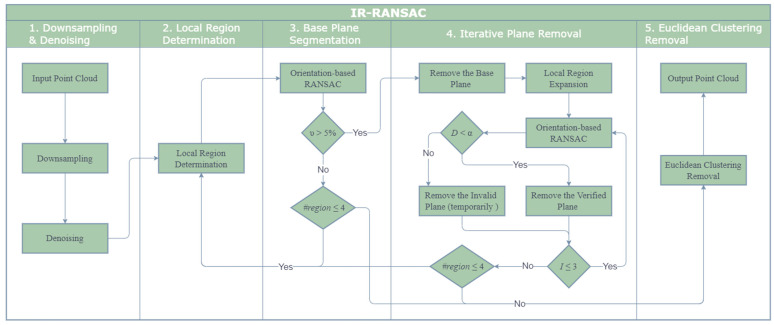
The flowchart of IR-RANSAC.

Our Iterative Region-based RANSAC (IR-RANSAC) has five main steps, as illustrated in Figure 1. We begin with two preprocessing techniques, first down-sampling the raw point cloud and then removing noisy or outlying points in the depth map. Second, we divide the point cloud space into four overlapped local regions based on the current view of the sensor. Third, we segment a base plane in each of the four local regions. Fourth, we implement an iterative plane removal technique to all four local regions, segmenting and removing the super-surfaces. Finally, we cluster the remaining point cloud using the geometric relationship between groups of points, resulting in a final point cloud comprised only of clustered objects of interest.

### 2.1. Downsampling and Denoising

Since input point clouds are generally large in size due to the significant number of 3D points and associated color information, a downsampling method with low computational complexity can significantly reduce the running time of point cloud processing algorithms. Downsampling is typically achieved using either a random downsample method [48] or a voxelized grid approach [49]. Although the former is more efficient, the latter preserves the shape of the point cloud better and exploits the geometric relationship of the underlying samples. Since we are predominantly concerned with preserving the underlying points that represent the true geometry of objects in the scene, we utilize a voxelized grid approach [49] that returns the centroid of all the points in each 3D voxel grid with a leaf size of 0.1 cm. In this way, the downsampled point clouds will still reflect the structure and maintain the geometric properties of the original point cloud while reducing the total amount of points that will need to be processed and stored.

**Figure 2:**
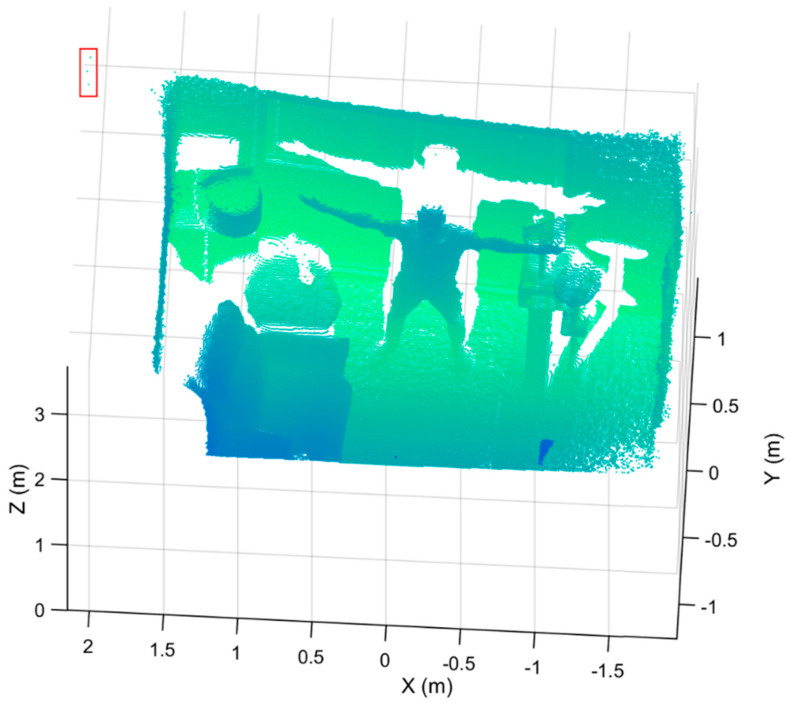
A sample point cloud with false data points (surrounded by a red rectangle), detected as the noise outside the true boundaries of the room. These noise artifacts artificially expand the overall outer dimensions of the point cloud.

Removing noisy points is a critical point cloud preprocessing task. Noisy or spurious points have two significant impacts on our approach. A noisy point cloud with false or spurious data points, including points outside of a scene’s real boundaries (see Figure 2 for example) can lead to a wrong measurement of the overall bounding box containing the point cloud, resulting in the definition of incorrect local regions in our subsequent processing steps. Furthermore, noisy points within the point cloud itself will effectively skew or change the geometry of the true objects. We utilize a statistical outlier removal approach [50] by examining the k-nearest neighbors (K=4) of each point, and removing all points with a distance (σ) of more than one standard deviation of the mean distance to the query point to remove outliers of each captured point cloud. If the average distance of a point to its k-nearest neighbors is above the threshold (σ), it is considered as an outlier. In this way, we remove points that are dissimilar from other points in their neighborhood. Together, these approaches decrease the number of points in the point cloud, reducing downstream processing time and increasing the accuracy of our process.

### 2.2. Local Region Determination

Dividing our captured point clouds into four local regions of interests, based on the properties of our indoor environments, reduces the possibility of detecting foreground planes, increases computational efficiency, and leverages the likely spatial location of potential bounding surfaces. In this way, we exploit knowledge of the scene based on the known sensor perspective, while allowing for surface locations to vary relative to each other in different rooms. Further, these regions help ensure that foreground objects that may appear planar in composition (e.g., tables, beds) are preserved and differentiated from background bounding surfaces.

**Figure 3:**
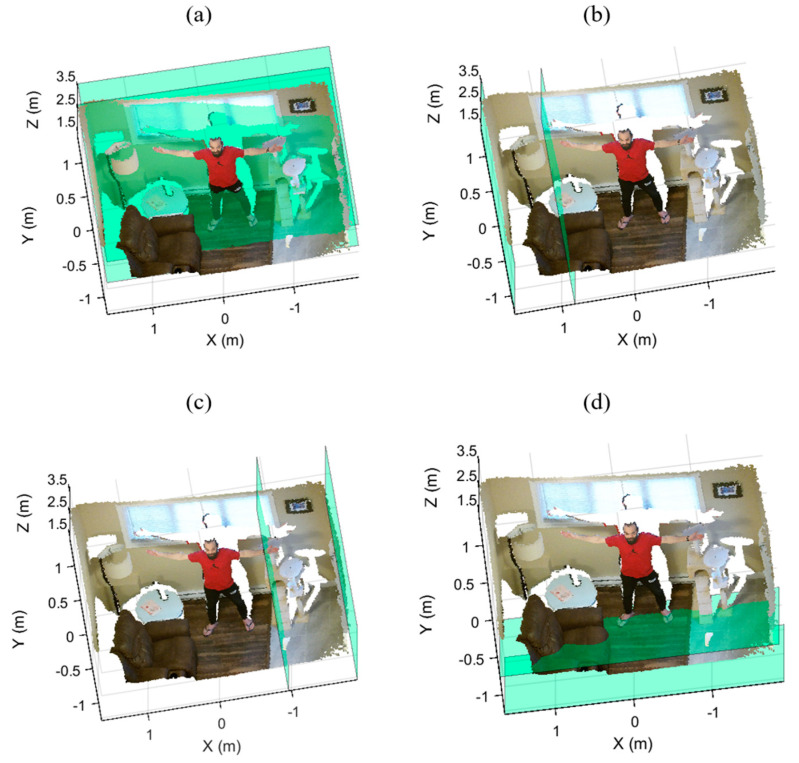
The boundaries of the four local regions highlighted in green: (**a**) the back, (**b**) left, (**c**) right, and (**d**) bottom regions.

We partition the point cloud space into four overlapped local regions based on the current view of the sensor. First, we find the bounding values of the downsampled and denoised point cloud, where the values $x_{min},x_{max,}y_{min},y_{max,}z_{min}$ and $z_{max}$ are the Euclidean extrema of the bounding box enclosing the point cloud, and $\left(x_{range},y_{range},z_{range}\right)=\left(x_{max},y_{max},z_{max}\right)-\left(x_{min},y_{min},z_{min}\right)$ are the Euclidean dimensions of the bounding box. Using the ranges defined in Table 1, we then determine the four local regions (see Figure 3 for a sample visualization of the local regions). We will use these four regions to identify, segment, and remove potential super-surfaces in each region.

Since our approach must be independent of any prior knowledge about the geometry of the indoor environment and both the location and perspective of the sensor, the four initial local regions may not include all the points that are actually part of the super-surfaces (e.g., Figure 3d, where parts of the floor are not included in within the local region).

Selecting larger initial regions will increase the likelihood that all true points are within the regions but will also increase the likelihood of including points belonging to foreground objects near the super-surfaces (e.g., beds and sofas). To resolve this issue, we implement conservative local regions and extend these four regions after base plane segmentation (see Section 2.4).

### 2.3. Base Plane Segmentation

We utilize the RANSAC algorithm [32] to segment the largest planes with a specific orientation in each of the local regions. All segmented plane candidates with more points than a learned value υ=5% of the total number of points in the point cloud, are verified as base planes and stored for use in the next step (Section 2.4). Planes containing fewer than υ points may be associated with key objects or small bounding planes and are dealt with in subsequent processing steps. Furthermore, υ is set as a proportion of the total points such that it is adaptive to the size of the point cloud.

The RANSAC algorithm iteratively and randomly samples three voxels, A, B and C as a minimum subset to generate a hypothesis plane. These three points represent two vectors $\vec{AB}$ and $\vec{AC}$, and their cross product is the normal vector $\vec{N}=\left(a,b,c\right)$ to the plane ax+by+cz+d=0. Therefore, the three parameters of the plane (a, b, and c) are computed, and d can be solved. In each iteration, the algorithm computes the distance $D=\left|\frac{ax+by+cz+d}{\sqrt{a^{2}+b^{2}+c^{2}}}\right|$ between all the remaining data points and the plane and then counts the number of points within a distance threshold (δ=4 cm) of the plane. Finally, RANSAC returns the plane with the highest percentage of inliers.

**Figure 4:**
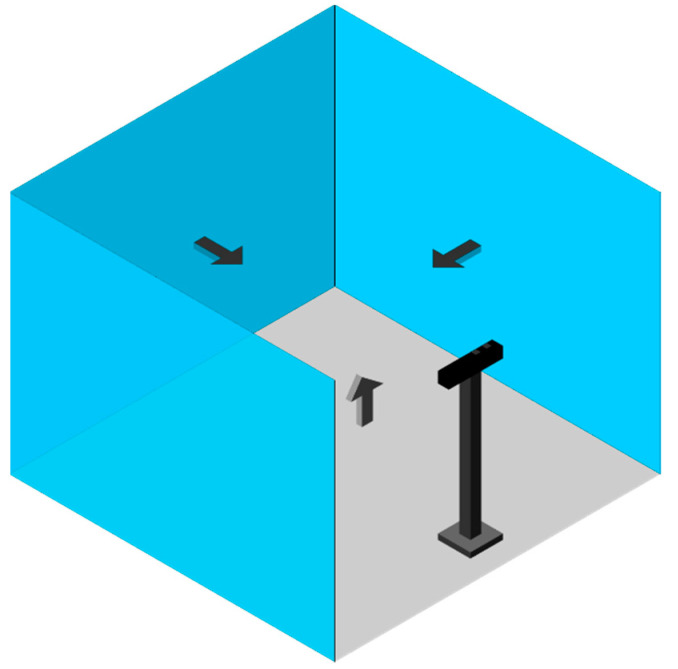
The initial reference vectors.

We add an orientation constraint to the standard RANSAC (orientation-based RANSAC) so that we assign priority to segmented planes with the highest percentage of inliers that have an expected orientation relative to the local regions. To do this, we defined an initial reference vector for each of the local regions, aligned with the sensor axes as [0,0,−1], [−1,0,0], [1,0,0] and [0,1,0] for the back, left, right, and bottom regions, respectively (Figure 4). Further, we define a maximum allowance angular variation (ω=45 degrees) between the normal vector of the planes and our reference vectors to allow for sensor perspective variations.

The maximum number of iterations T required for convergence by the RANSAC algorithm can be approximated as Equation (1) [51]. Convergence depends on the number of samples s (s=3 for the plane fitting), the target success probability p (e.g., p=99%), and the outlier ratio e. Considering there is no prior knowledge about the underlying outlier ratio, it is difficult to approximate the number of RANSAC iterations. Based on our experimental results and due to the iterative design of IR-RANSAC, the algorithm works appropriately with the number of trials adhering to 1%≤T≤2% of the data points within each local region. In this study, we set T to 2% of the data points (e.g., if the back region contains 60,000 points, the maximum number of trials will be set to 1200). Increasing p and T improve the robustness of the output at the expense of additional computation:

(1) $$T=\frac{\log\left(1-p\right)}{\log\left(1-{\left(1-e\right)}^{s}\right)}$$

### 2.4. Iterative Plane Removal

In a complex indoor environment, bounding surfaces such as walls, windows, and curtains are difficult to fit to a single plane. Increasing the distance threshold (δ) includes more points near the bounding planes, but simultaneously increases the chance of including data from important objects (e.g., the human body) within the extended threshold. Furthermore, the input point cloud can be unorganized, which means the nearest neighbor operations, such as region growing, are not very efficient for segmenting the rest of the super-surfaces. We introduce a novel iterative plane removal technique to segment and remove super-surfaces from a point cloud while minimizing the likelihood of including points that belong to foreground objects.

First, we remove the verified base planes associated with each local region. Then, we expand the local regions according to the ranges in Table 2 to completely encompass the areas containing the super-surfaces. Next, we apply the orientation-based RANSAC in each of the extended regions iteratively. The number of iterations depends on the complexity of the indoor environment; based on our experimental results, three iterations are adequate for a challenging indoor environment. In each iteration, segmented planes must be parallel to the base plane of the current region. Hence, we utilize the normal vectors of the base planes as the reference vectors, and we set the maximum allowance angular variation (θ) to 5°. Finally, because employing the orientation-based RANSAC in a larger region increases the probability of a false segmentation, we validate the segmented planes in each iteration.

The segmented planes are validated based on their distances, *D*, from their base planes, where a, b, c, and d are parameters of the base plane, and x, y and z are coordinates of a point on the segmented plane. To make the technique robust to high levels of noise, we substitute the distance of a point to the base plane with the mean of all the segmented points’ distances from the base plane.

**Figure 5:**
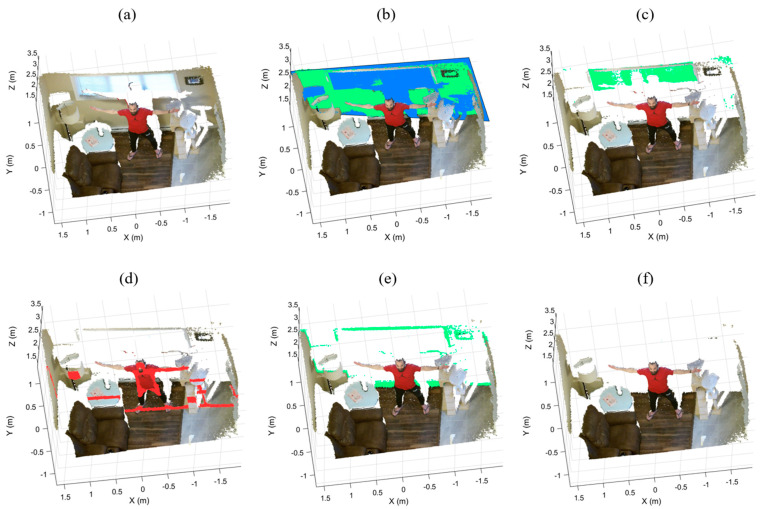
The iterative plane removal of the back region: (**a**) the sample point cloud, (**b**) the verified base plane, (**c**,**e**) the verified segmented planes, (**d**) the invalid segmented plane, and (**f**) the remaining point cloud after the back wall removal.

If the distances are less than a threshold (e.g., α=10 cm), those planes will be removed from their regions. Otherwise, they are not part of the super-surfaces and will be temporarily removed from the remaining point cloud. There are two advantages to temporarily removing a false segmented plane. First, it prevents RANSAC from segmenting the false plane once again. Second, it reduces the current region for the next iteration. Figure 5 illustrates the output of the iterative plane removal in each iteration when applied to the back region of a point cloud. The green planes are verified and eliminated from the back region, as shown in Figure 5b,c,e. However, the segmented red plane is not verified and temporarily removed from the point cloud, as shown in Figure 5d.

### 2.5. Euclidean Clustering Removal

**Figure 6:**
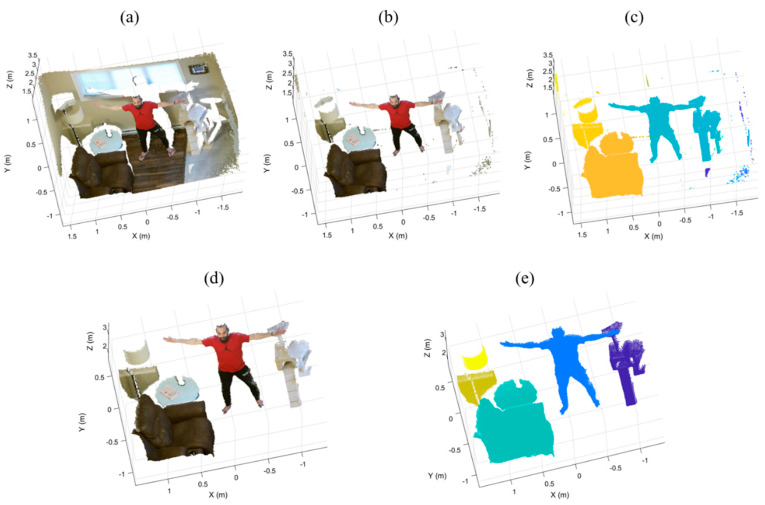
Euclidean clustering on the sample point cloud (**a**) following the iterative plane removal (**b**) results in properly segmented foreground object clusters as well as residual clusters (**c**). Removing object clusters with fewer than μ points leaves only foreground objects (**d**) which can be visualized as colored objects (**e**).

In this step, we cluster the remaining point cloud based on Euclidean distance to remove the irrelevant small segments and keep the objects of interest. First, we compute the Euclidean distance between each point and its neighbors. Then, we group neighboring points as a cluster if the distance between any point in an object and an adjacent point is less than a threshold ε=5 cm, finishing when all the clusters are determined. Finally, we remove all small clusters with fewer than a threshold μ=500 points. Figure 6 illustrates an example of Euclidean clustering removal following the iterative plane removal.

## 3. Experiments and Evaluation

### 3.1. Experimental Setup

**Figure 7:**
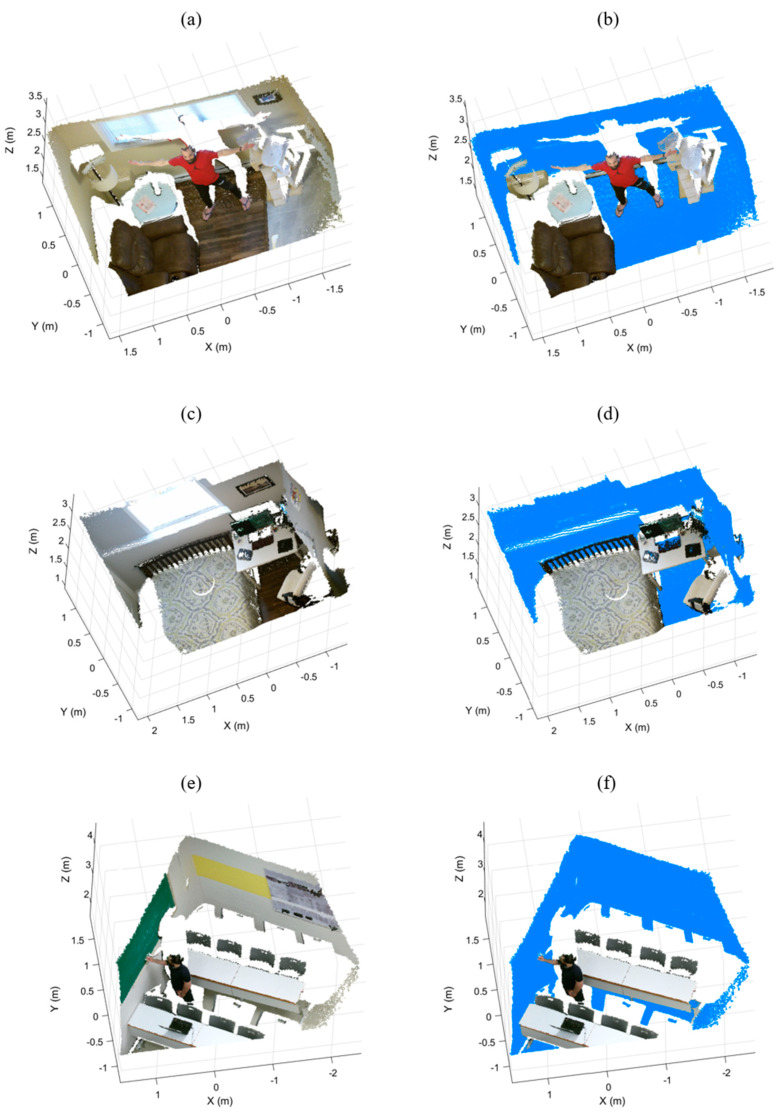
The generated datasets, Room-1 (**a**), Room-2 (**c**), and Room-3 (**e**), and their manually labeled super-surfaces in blue color, (**b**), (**d**), and (**f**) respectively.

We evaluated our method on three generated datasets representing three different complex indoor environments, Room-1, Room-2, and Room-3, as shown in Figure 7a,c,e respectively. Each dataset contains different objects, such as furniture, planar objects, and human bodies. To measure the performance of IR-RANSAC in our challenging environments, we acquired each point cloud from an arbitrary oblique-view location using the Microsoft Kinect V2 sensor. The details of each dataset after the preprocessing steps are listed in Table 3.

To define the ground truth, we manually labeled the points belonging to each super-surface in every dataset by deploying the MATLAB Data tips tool based on their coordinates, colors, and the super-surface definition. We employed this lengthy and precise procedure by first using a semi-automated labeling approach using the orientation-based RANSAC and MATLAB Data tips tool. First, we segmented as many super-surface points as possible by running the orientation-based RANSAC in manually selected local regions known to contain super-surfaces. Then, we modified and validated the previous labeled points resulting in our final manually labeled super-surfaces shown in Figure 7b,d,f.

We evaluated the efficiency of our IR-RANSAC and RANSAC (as a baseline) in terms of four pixel-based metrics, precision, recall, F_1_ score, and specificity. The first three parameters have been widely utilized for appraising the effectiveness of plane segmentation (e.g., [36,52,53]). To compute these metrics, we defined true positive (TP) as bounding surface points correctly identified, true negative (TN) as foreground points correctly identified, false positive (FP) as foreground points incorrectly identified as bounding surface points, and false negative (FN) as bounding surface points incorrectly identified as foreground points. Precision, measured as $\frac{TP}{TP+FP}\;$, is the number of correctly removed points (i.e., true positives) with respect to the total number of removed points. Recall, measured as $\frac{TP}{TP+FN}\;$, is the fraction of true positive among the manually labeled points (ground truth). F_1_ score, measured as $2\times \frac{precision\times recall}{precision+recall}\;$, or the harmonic mean of the precision and recall, represents the overall performance of our proposed method. The specificity, measured as $\frac{TN}{TN+FP}\;$, reflects the true negative rate of our algorithm, providing a measure of how well our method distinguishes between foreground points and bounding surface points.

We computed the size reduction of IR-RANSAC as $1-\frac{S_{OUT}}{S_{IN}}$ where $S_{OUT}$ and $S_{IN}$ are the size (i.e., the number of points) of the output point cloud and the input point cloud, respectively. We implemented our proposed algorithm running MATLAB on an Intel i5-4300M CPU @ 2.60 GHz and with 6.00 GB RAM. The full parameters of IR-RANSAC, determined through experimentation, are listed in Table 4 and used for all our experiments.

### 3.2. Experimental Results

**Figure 8:**
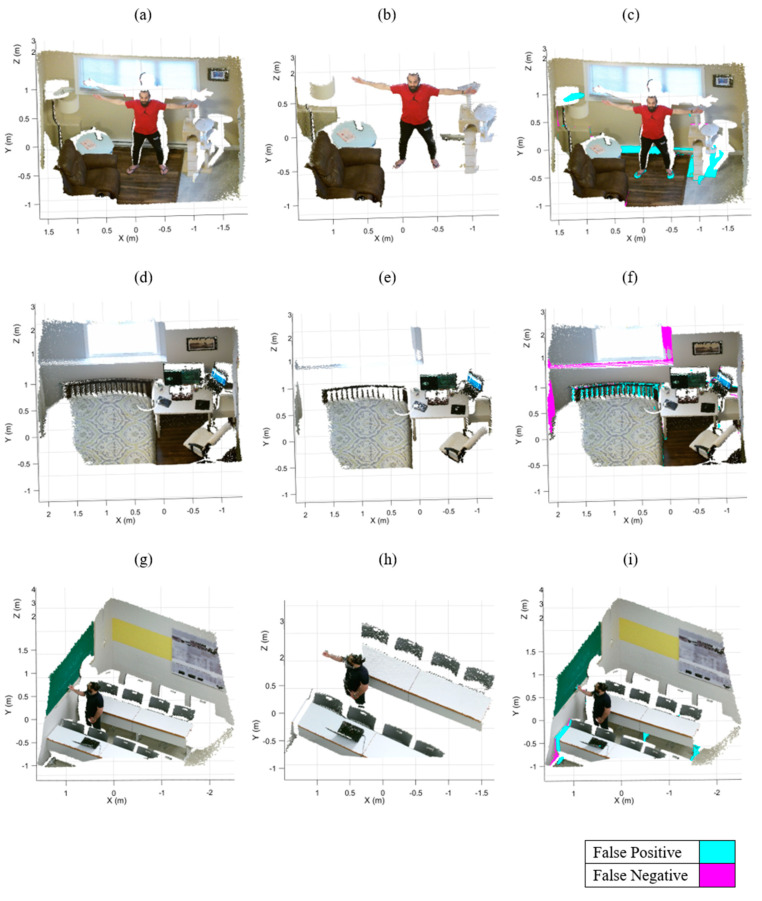
Output results of IR-RANSAC. The original point clouds, Room-1 (**a**), Room-2 (**d**), and Room-3 (**g**), are visualized with all super-surfaces removed (**b**), (**e**), and (**h**) respectively. The false positive (cyan) and false negative (magenta) points are highlighted over the original point clouds for the Room-1 (**c**), Room-2 (**f**) and Room-3 (**i**) datasets.

Figure 8 shows the output results of our algorithm and erroneously classified points for the three datasets.

Incorrectly classified points are almost always associated with the points belonging to foreground objects that are contacting a super-surface (i.e., objects within the RANSAC distance threshold), such as the baseboard heater, the bed headboard, and the desk legs (Figure 8c,f,i respectively). Notably, as a result of our local region definitions and bounding surface criteria, we successfully prevented consideration of foreground objects that could be misidentified as bounding surfaces (e.g., beds, desks). Furthermore, foreground objects comprised of fewer points than the Euclidean clustering threshold (μ=500 points) were erroneously removed from the point cloud (e.g., the cyan portion of the lamp in Figure 8c).

Our IR-RANSAC method eliminated nearly all points belonging to the super-surfaces from the Room-1 and Room-3 datasets. However, IR-RANSAC failed to remove two small challenging regions belonging to the back and left super-surfaces of Room-2 (the magenta regions around the window and the bottom left corner of the point cloud in Figure 8f). This is because the planes surrounding the window are perpendicular to the back super-surface, and the other small plane in the bottom left corner is smaller than the verification threshold (υ=5% of the total number of points in the point cloud). Notably, both of these two miss-classified regions are greater than the Euclidean clustering threshold (μ=500 points). Additionally, IR-RANSAC incorrectly assigned a small number of points belonging to foreground objects to a bounding surface. In all cases, these incorrect assignment of foreground points to super-surfaces happened when clustered foreground objects physically contacted super-surfaces (e.g., bed headboard in Figure 8f and desk legs in Figure 8i).

**Figure 9:**
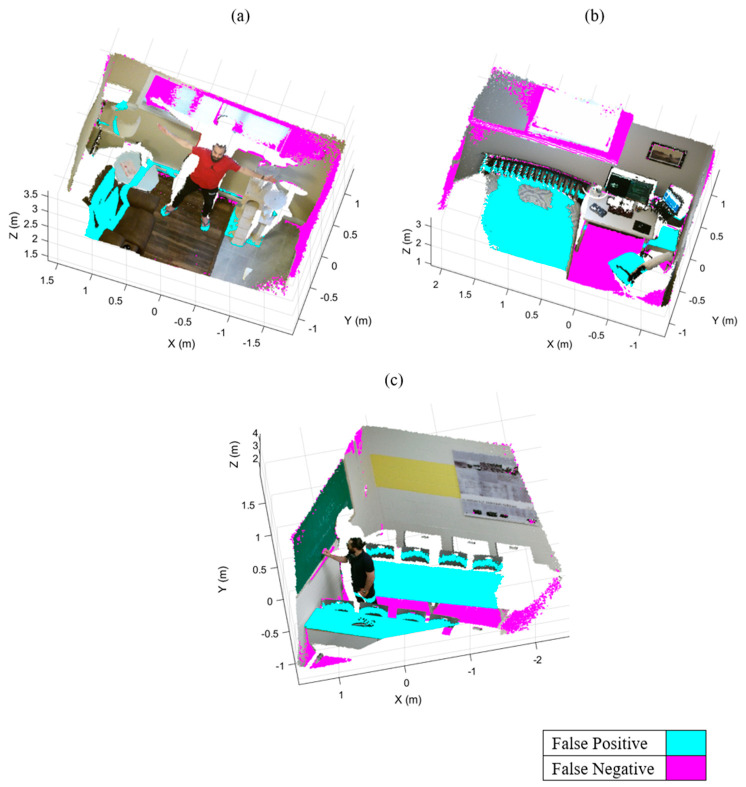
The output results of standard RANSAC plane removal and its false positives and false negatives in cyan and magenta, respectively: (**a**) the Room-1, (**b**) the Room-2, and (**c**) the Room-3.

Figure 9 shows the output results of the standard RANSAC plane removal for the three datasets. The cyan and magenta colors represent the incorrectly removed regions (i.e., false positive) and undetected regions (i.e., false negative), respectively. The standard RANSAC approach failed to remove many points belonging to the super-surfaces (e.g., the back and right walls of Room-1 and the floors of the other two datasets). Moreover, RANSAC erroneously removed some parts of the human body and the furniture (e.g., couch, bed, and desks). These false segmentations have three main reasons: RANSAC sensitivity to clutter and occlusion, the uncertainty of RANSAC in randomly sampling three points as a minimum subset, and the lack of an orientation constraint.

Our experimental results suggest that IR-RANSAC supports varied sensor locations and removes background boundary surfaces more effectively without removing foreground data in complex 3D indoor environments.

### 3.3. Evaluation

**Figure 10:**
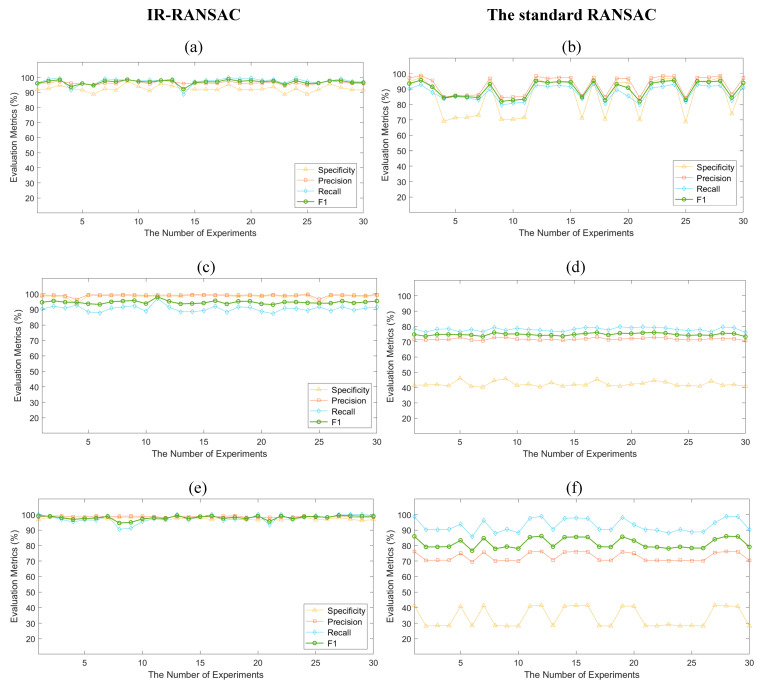
The evaluation results of IR-RANSAC and the standard RANSAC plane removal for the three datasets: (**a**,**b**) the Room-1, (**c**,**d**) the Room-2, (**e**,**f**) the Room-3.

Similar to RANSAC, our IR-RANSAC is stochastic, and accordingly its results can vary depending on the selection of the random subsample. To account for this stochasticity, we conducted 30 experiments on each dataset, similar to the work of Li et al. [39], and computed our four evaluation metrics for IR-RANSAC and standard RANSAC plane removal as a baseline comparator. We illustrate the evaluation results in Figure 10, with the left column visualizing our IR-RANSAC results and the right column representing our standard RANSAC plane removal results, and the rows corresponding to the three rooms. We implemented the standard RANSAC plane removal traditionally to segment and eliminate the four largest planes in each dataset. We set the parameters of our benchmark RANSAC method to be the same as those used in IR-RANSAC. The mean (M) and standard deviation (SD) of both approaches are shown in Table 5 for specificity, precision, recall, and F_1_ score, along with execution times.

Given the original size of the point clouds (see Table 3), and the size of the output point clouds for Room-1 (55,705 points), Room-2 (69,044 points), and Room-3 (66,149 points), IR-RANSAC yielded a size reduction of 0.68, 0.62, and 0.64 for Room-1, Room-2, and Room-3, respectively.

## 4. Discussion

Our evaluation results achieved with IR-RANSAC (the first column of Figure 10) are higher and much more consistent in all the four evaluation metrics than those obtained using standard RANSAC (the second column of Figure 10). Additionally, almost all four scores have a lower standard deviation with IR-RANSAC compared to standard RANSAC. Overall, all of our evaluation results were statistically significantly better (p<0.05) with IR-RANSAC than standard RANSAC using a two-sample *t*-test. Most notably, the F_1_ score, which represents the overall performance of the approaches, was statistically higher with IR-RANSAC than standard RANSAC.

In all experiments, our proposed IR-RANSAC method obtained average values above 92% for specificity, 96% for precision, 90% for recall, and 94% for F_1_ score. Comparably, the standard RANSAC achieved average values between 33% and 85%, 71% and 92%, 78% and 92%, 74% and 90% for specificity, precision, recall, and F_1_ score, respectively. As illustrated in the second column of Figure 10, there are also many sharp fluctuations in the standard RANSAC evaluation results. The F_1_ score fluctuated from 82% to 95% and 76% to 86% for Room-1 and Room-3, respectively. However, it almost remained steady at 74% for the Room-2 dataset. The standard RANSAC approach demonstrated a very low specificity for Room-2 and Room-3, containing many planer furniture.

IR-RANSAC takes about three times as long to execute when compared to the standard RANSAC approach on the same datasets. This is expected though, since our IR-RANSAC method invokes the RANSAC algorithm four times more than the standard RANSAC plane removal. Theoretically, a faster version of IR-RANSAC, which has only one iteration in each local region, can be implemented that would be faster than the standard RANSAC approach because it runs the RANSAC algorithm in smaller regions.

Our evaluation results support that IR-RANSAC is a robust and reliable method for removing the bounding super-surfaces of a complex 3D indoor environment with better performance over traditional RANSAC in all ways except execution time. Our results suggest that IR-RANSAC removes background boundary surfaces effectively without removing foreground data, and can considerably reduce size of 3D point clouds.

The subjects of future research are speeding up the IR-RANSAC algorithm and improving its results in much more complex 3D indoor environments. To improve our algorithm results, we need to reduce its reliance on the Euclidean clustering technique, eliminate the small challenging regions belonging to a super-surface but with a different normal vector (e.g., the highlighted regions around the window in Figure 8f), and implement self-adaptive parameters to be robust to different indoor environments and sensor data.

## 5. Conclusions

We have presented a 3D bounding surface removal technique, IR-RANSAC, that is particularly suited to more challenging and cluttered indoor environments. IR-RANSAC supports varied sensor perspectives for organized and unorganized point clouds, and it considerably reduces the size of 3D datasets. Moreover, IR-RANSAC can improve the results and the running time of different 3D computer vision methods by reducing their search space. After downsampling and denoising a point cloud captured from an oblique view, we divide the point cloud space into four overlapped local regions, exploiting knowledge of the current view of the sensor, and segment a base plane in each of the four regions. We then expand our search space around the base plane in each region, and iteratively segment and remove the remaining points belonging to each super-surface. Finally, we cluster the remaining point cloud using the geometric relationship between groups of points, resulting in a final point cloud comprised only of clustered objects of interest. We evaluated the performance of IR-RANSAC in terms of four metrics: specificity, precision, recall, and F1 score, on the three generated datasets acquired from an arbitrary oblique-view location and representing different indoor environments. Our experiments demonstrated that our proposed method is a robust super-surface removal and size reduction technique for complex 3D indoor environments. Experimentally, IR-RANSAC outperformed traditional RANSAC segmentation in all categories, supporting our efforts to prioritize the inclusion of all bounding points in each super-surface, while minimizing inclusion of points that belong to foreground objects.

Our intention was to develop a robust method of bounding surface segmentation—maximizing inclusion of bounding surface points and minimizing inclusion of foreground points. Our experimental data suggest that by conceptualizing bounding surfaces (e.g., walls and floor) as unique and different than other large surfaces that belong to foreground objects, it is possible to improve on methods of segmenting and removing these unwanted bounding surfaces specifically. By removing these bounding surfaces and preserving foreground objects, we considerably reduce the size of the resulting dataset, substantially improving downstream storage and processing.

## Figures and Tables

**Table 1:** Ranges for each local region.

Region	Range		
	X-Axis	Y-Axis	Z-Axis
Back	(−∞,∞)	(−∞,∞)	$\left[z_{max}-\left(\frac{z_{range}}{4}\right),\;z_{max}\right]$
Left	$\left[x_{max}-\left(\frac{x_{range}}{4}\right),\;x_{max}\right]$	(−∞,∞)	(−∞,∞)
Right	$\left[x_{min},\;x_{min}+\left(\frac{x_{range}}{4}\right)\right]$	(−∞,∞)	(−∞,∞)
Bottom	(−∞,∞)	$\left[y_{min},y_{min}+\left(\frac{y_{range}}{4}\right)\right]$	(−∞,∞)

**Table 2:** Extended ranges for each local region.

Region	Range		
	X-Axis	Y-Axis	Z-Axis
Back	(−∞,∞)	(−∞,∞)	$\left[z_{max}-\left(\frac{z_{range}\times 3}{4}\right),\;z_{max}\right]$
Left	$\left[x_{max}-\left(\frac{x_{range}}{2}\right),\;x_{max}\right]$	(−∞,∞)	(−∞,∞)
Right	$\left[x_{min},\;x_{min}+\left(\frac{x_{range}}{2}\right)\right]$	(−∞,∞)	(−∞,∞)
Bottom	(−∞,∞)	$\left[y_{min},y_{min}+\left(\frac{y_{range}}{2}\right)\right]$	(−∞,∞)

**Table 3:** Parameters of the datasets.

Description	Width (m)	Height (m)	Depth (m)	Number of Points
**Room-1**	3.56	2.65	3.68	179,572
**Room-2**	3.51	2.60	3.46	179,374
**Room-3**	4.11	3.27	4.71	181,822

**Table 4:** Parameters of IR-RANSAC.

Procedure	Descriptor	Parameter	Value
**Denoising algorithm**	Nearest neighbors	*K*	4
	Outlier threshold	σ	1 standard deviation
**Base plane segmentation**	RANSAC distance threshold	δ	4 cm
	Maximum allowance angular variation	ω	45°
	RANSAC maximum iterations	*T*	2% of region points
	Verification threshold	υ	5% of point cloud points
**Iterative plane removal**	Iterations	*I*	3
	Maximum allowance angular variation	θ	5°
	Distance threshold between two planes	α	10 cm
**Euclidean clustering removal**	Euclidean clustering threshold	μ	500 points
	Euclidean distance threshold	ε	5 cm

**Table 5:** Execution times, mean (M), and standard deviation (SD) of IR-RANSAC and standard RANSAC for specificity, precision, recall, and F_1_ score.

Dataset	Method	Specificity		Precision		Recall		F_1_		Runtime (s)
		M (%)	SD	M (%)	SD	M (%)	SD	M (%)	SD	
**Room-1**	IR-RANSAC	92.60	0.0200	96.41	0.0097	97.42	0.0230	96.90	0.0142	8.83
	Standard RANSAC	85.47	0.1210	92.56	0.0612	87.60	0.0484	90.00	0.0538	3.20
**Room-2**	IR-RANSAC	98.38	0.0135	99.06	0.0074	90.52	0.0193	94.59	0.0101	7.50
	Standard RANSAC	42.37	0.0164	71.79	0.0067	78.10	0.0114	74.81	0.0077	2.80
**Room-3**	IR-RANSAC	97.39	0.0075	98.61	0.0038	97.47	0.0253	98.02	0.0126	6.37
	Standard RANSAC	33.91	0.0644	72.72	0.0278	92.78	0.0419	81.53	0.0333	2.40

## Data Availability

The data presented in this study are available on request from the corresponding author. The data are not publicly available at the time of publication due to insufficient resources for making the data publicly accessible.
