# Correction: Ebrahimi, A.; Czarnuch, S. Automatic Super-Surface Removal in Complex 3D Indoor Environments Using Iterative Region-Based RANSAC. *Sensors* 2021, *21*, 3724

**DOI:** 10.3390/s24227176

**Published:** 2024-11-08

**Authors:** Ali Ebrahimi, Stephen Czarnuch

**Affiliations:** 1 Department of Electrical and Computer Engineering, Faculty of Engineering and Applied Science, Memorial University of Newfoundland, St. John’s, NL A1C 5S7, Canada; 2 Discipline of Emergency Medicine, Faculty of Medicine, Memorial University of Newfoundland, St. John’s, NL A1C 5S7, Canada

**Affiliation Correction**

In the original publication [1], the second author’s email address has been removed from Affiliation 1.

**Text Correction**

1. Abstract. The word “floor” has been updated to “floors”; the phrase “association of foreground points to” has been updated to “association of foreground points with ”; the phrase “real-world scene ” has been updated to “real-world scenes”; the phrase “3D complex indoor environments” has been updated to “complex 3D indoor environments”; the word “evaluate” has been updated to “evaluated”; the sentence “while scoring the four evaluation metrics between 90% and 99%.” has been updated to “with scores of the four evaluation metrics ranging between 90% and 99%.”.

2. Section 1. In first paragraph, the phrase “As /a result” has been updated to “As a result”. In third paragraph, the phrase “improvements that to our knowledge” has been updated to “these improvements, to our knowledge,”. In fourth paragraph, the phrase “and curtains” has been updated to “or curtain”.

3. Section 1.1. The phrase “where indexes are referenced” has been updated to “where indices are referenced”. The sentence “While it is trivial to convert an organized to an unorganized point cloud, the converse is much more complicated and costly.” has been updated to “While it is trivial to convert an organized point cloud to an unorganized point cloud, the reverse is much more complicated and costly.”. The phrase “as derived from” has been updated to “derived from”.

4. Section 1.1.1. In second paragraph, the word “Terminally” has been updated to “Finally”. In third paragraph, the phrase “planer surfaces” has been updated to “planar surfaces”; the phrase “region growing based ” has been updated to “region growing-based”.

5. Section 1.1.2. In first paragraph, the phrase “normal vectors estimation” has been updated to “normal vector estimation”. In second paragraph, the phrase “intended for only” has been updated to “only intended for”. In second paragraph, the word “was” has been updated to “is” and “refined” has been updated to “refines”.

6. Section 1.1.3. In first paragraph, “Filin [40]” has been updated to “Filin [48]”; the phrase “Filin and Pfeifer [41] computed the” has been updated to “Filin and Pfeifer [49] computed”; the phrase “merged those clusters” has been updated to “merged these clusters”; the phrase “if they share” has been updated to “if they shared”; the phrase “Czerniawski et al. [21] ” has been updated to “Czerniawski et al. [28]”; the phrase “Zhou et al. [42]” has been updated to “Zhou et al. [50]”; the phrase “using planes’ normal vectors” has been updated to “using the planes’ normal vectors”; the phrase “planer surfaces” has been updated to “planar surfaces”. In paragraph 2, “[28]” has been updated to “[36]”; “[43]” has been updated to “[51]”.

7. Section 2. The phrase “Our Iterative Region-based RANSAC (IR-RANSAC)” has been updated to “Our iterative region-based RANSAC (IR-RANSAC) method”.

8. Section 2.1. In first paragraph, the phrase “a random downsample method [48] or a voxelized grid approach [49]” has been updated to “a random downsampling method [52] or a voxelized grid approach [53]”; the phrase “we utilize a voxelized grid approach [49] that” has been updated to “we utilized a voxelized grid approach [53] that”. In second paragraph, the phrase “We utilize a statistical outlier removal approach [50] by” has been updated to “We utilized a statistical outlier removal approach [54] by”; the phrase “reducing downstream processing time” has been updated to “reducing the downstream processing time”.

9. Section 2.2. In third paragraph, the phrase “included in within” has been updated to “included within”.

10. Section 2.3. In third paragraph, the phrase “the standard RANSAC” has been updated to “the standard RANSAC algorithm”. In fourth paragraph, “[51]” has been updated to “[55]”; “improve” has been updated to “improves”.

11. Section 2.4. In fourth paragraph, the phrase “those planes ” has been updated to “the planes”.

12. Section 3.1. In first paragraph, the phrase “using the Microsoft Kinect V2 sensor” has been updated to “using a Microsoft Kinect V2 sensor”. In second paragraph, the sentence “Then, we modified and validated the previous labeled points resulting in our final manually labeled super-surfaces shown in Figure 7b,d,f.” has been updated to “Then, we modified and validated the previously labeled points, resulting in the final manually labeled super-surfaces shown in Figure 7b,d,f.”.

13. Caption of Figure 7. The phrase “blue color, (**b**), (**d**), and (**f**) respectively.” has been updated to “blue (**b**), (**d**), and (**f**), respectively.”

14. The correct Paragraphs 3 and 4 of Section 3.1 appear below:

We evaluated the efficiency of our IR-RANSAC and RANSAC (as a baseline) methods in terms of four pixel-based metrics: precision, recall, F_1_ score, and specificity. The first three parameters have been widely utilized for appraising the effectiveness of plane segmentation (e.g., [36,51,56]). To compute these metrics, we defined a true positive (TP) as a correctly identified bounding surface point, a true negative (TN) as a correctly identified foreground point, a false positive (FP) as a foreground point that was incorrectly identified as a bounding surface point, and a false negative (FN) as a bounding surface point that was incorrectly identified as a foreground point. Precision, calculated as $\frac{TP}{TP+FP}$, is the number of correctly removed points (i.e., true positives) with respect to the total number of removed points. Recall, calculated as $\frac{TP}{TP+FN}$, is the fraction of true positives among the manually labeled points (ground truth). F_1_ score, calculated as $2\times \frac{precision\times recall}{precision+recall}$, or the harmonic mean of the precision and recall, represents the overall performance of our proposed method. The specificity, calculated as $\frac{TN}{TN+FP}$, reflects the true negative rate of our algorithm, providing a measure of how well our method distinguishes between foreground points and bounding surface points.

We computed the size reduction of IR-RANSAC as $1-\frac{S_{OUT}}{S_{IN}}$, where $S_{OUT}$ and $S_{IN}$ are the sizes (i.e., the number of points) of the output point cloud and the input point cloud, respectively. We implemented our proposed algorithm using MATLAB on an Intel i5-4300M CPU @ 2.60 GHz and with 6.00 GB RAM. The full parameters of IR-RANSAC, determined through experimentation, are listed in Table 4 and were used for all our experiments.

15. Section 3.2. In first paragraph, the phrase “algorithm and erroneously” has been updated to “algorithm and the erroneously”. In second paragraph, the phrase “Incorrectly classified points are ” has been updated to “The incorrectly classified points were”. In third paragraph, the phrase “super-surfaces from the Room-1” has been updated to “super-surfaces in the Room-1”; the phrase “super-surfaces of Room-2” has been updated to “super-surfaces in Room-2”; the phrase “window are perpendicular to” has been updated to “window were perpendicular to”; the phrase “corner is smaller than” has been updated to “corner was smaller than”; the phrase “two miss-classified regions” has been updated to “two misclassified regions”; the phrase “happened when” has been updated to “happened in cases where the”. In fourth paragraph, the phrase “removal for” has been updated to “removal approach for”; the phrase “right walls of Room-1 and the floors of the” has been updated to “right walls in Room-1 and the floors in the”; the phrase “RANSAC sensitivity” has been updated to “RANSAC’s sensitivity”.

16. Caption of Figure 8. The phrase “removed (**b**), (**e**), and (**h**) respectively.” has been updated to “removed in (**b**), (**e**), and (**h**), respectively.”.

17. Caption of Figure 9. The phrase “(**a**) the Room-1, (**b**) the Room-2, and (**c**) the Room-3.” has been updated to “(**a**) Room-1, (**b**) Room-2, and (**c**) Room-3.”.

18. Section 3.3. In first paragraph, the phrase “our IR-RANSAC is stochastic,” has been updated to “our IR-RANSAC approach is stochastic,”; the phrase “ our standard RANSAC” has been updated to “the standard RANSAC”; the phrase “We implemented the standard RANSAC” has been updated to “We implemented standard RANSAC”; the sentence “The mean (M) and standard deviation (SD) of both approaches are shown in Table 5 for specificity, precision, recall, and F_1_ score, along with execution times.” has been updated to “The mean (M) and standard deviation (SD) of the specificity, precision, recall, and F_1_ score of both approaches are shown in Table 5, along with the execution times.”.

19. The correct Figure 10 caption appears below:

**Figure 10.** The evaluation results of IR-RANSAC and standard RANSAC plane removal for the three datasets: (**a**) and (**b**) Room-1, (**c**) and (**d**) Room-2, (**e**) and (**f**) Room-3.

20. The correct Table 5 caption appears below:

**Table 5.** Execution times, and mean (M) and standard deviation (SD) of specificity, precision, recall, and F_1_ score of IR-RANSAC and standard RANSAC methods.

21. Section 4. The correct Paragraph 1 appears below:

Our evaluation results for all four evaluation metrics achieved with IR-RANSAC (the first column of Figure 10) were higher and much more consistent than those obtained using standard RANSAC (the second column of Figure 10). Additionally, almost all four scores for IR-RANSAC had a lower standard deviation compared to those of standard RANSAC. Overall, all of our evaluation results for IR-RANSAC were statistically significantly better (p<0.05) than those of standard RANSAC using a two-sample t-test. Most notably, the F_1_ score, which represents the overall performance of the approaches, for IR-RANSAC was higher than that of standard RANSAC.

22. Section 4. In Paragraph 2, the phrase “planer furniture” has been updated to “planar furniture”. In Paragraph 5, the phrase “to be robust” has been updated to “to make the algorithm robust”.

**Funding Correction**

The word “Grant” has been updated to “grant number”.

**References Correction**

Three new References [48–50] have been added:

48 Filin, S. Surface clustering from airborne laser scanning data. In Proceedings of the International Archives of the Photogrammetry, Remote Sensing and Spatial Information Sciences, Denver, CO, USA, 10–15 November 2002; pp. 119–124.
49 Filin, S.; Pfeifer, N. Segmentation of airborne laser scanning data using a slope adaptive neighborhood. *ISPRS J. Photogramm. Remote Sens.* **2005**, *60*, 71–80. https://doi.org/10.1016/j.isprsjprs.2005.10.005.
50 Zhou, G.; Cao, S.; Zhou, J. Planar Segmentation Using Range Images From Terrestrial Laser Scanning. *IEEE Geosci. Remote Sens. Lett.* **2016**, *13*, 257–261. https://doi.org/10.1109/lgrs.2015.2508505.

The previous Reference [53] has been reordered as Reference [51]. With this correction, the order of some references has been adjusted accordingly. 

The authors state that the scientific conclusions are unaffected. This correction was approved by the Academic Editor. The original publication has also been updated.
